# Vision-Aided Velocity Estimation in GNSS Degraded or Denied Environments

**DOI:** 10.3390/s26030786

**Published:** 2026-01-24

**Authors:** Pierpaolo Serio, Andrea Dan Ryals, Francesca Piana, Lorenzo Gentilini, Lorenzo Pollini

**Affiliations:** 1 Department of Information Engineering, University of Pisa, 56127 Pisa, Italy; andrea.ryals@phd.unipi.it (A.D.R.); lorenzo.pollini@unipi.it (L.P.); 2 Research & Development, Toyota Material Handling Manufacturing, 40132 Bologna, Italy; lorenzo.gentilini@toyota-industries.eu

**Keywords:** navigation, GNSS degraded, camera, LiDAR, sensor fusion, state estimation

## Abstract

This paper introduces a novel architecture for a navigation system that is designed to estimate the position and velocity of a moving vehicle specifically for remote piloting scenarios where GPS availability is intermittent and can be lost for extended periods of time. The purpose of the navigation system is to keep velocity estimation as reliable as possible to allow the vehicle guidance and control systems to maintain close-to-nominal performance. The cornerstone of this system is a landmark-extraction algorithm, which identifies pertinent features within the environment. These features serve as landmarks, enabling continuous and precise adjustments to the vehicle’s estimated velocity. State estimations are performed by a Sequential Kalman filter, which processes camera data regarding the vehicle’s relative position to the identified landmarks. Tracking the landmarks supports a state-of-the-art LiDAR odometry segment and keeps the velocity error low. During an extensive testing phase, the system’s performance was evaluated across various real word trajectories. These tests were designed to assess the system’s capability in maintaining stable velocity estimation under different conditions. The results from these evaluations indicate that the system effectively estimates velocity, demonstrating the feasibility of its application in scenarios where GPS signals are compromised or entirely absent.

## 1. Introduction

In remote operations, where the pilot commands consist in velocity references for the vehicle controller, estimating precise velocity is crucial for accurate control and safety. The Global Navigation Satellite System (GNSS) is widely regarded as the gold standard for outdoor positioning and navigation. In environments with an unobstructed sky, satellite-derived position estimates facilitate effortless and precise navigation and velocity estimation. However, this positioning system exhibits several limitations when the context changes. Structures such as tall buildings, tunnels, or dense forests can obstruct communications with satellites, resulting in inaccurate or loss of location data. When transitioning between environments, such as moving from outdoor to indoor settings, navigation sensors typically experience discontinuities and significant changes in the characteristics of the signals they receive. Therefore, the data fusion module should be designed to accommodate these challenges, facilitating a seamless transition between the most informative sensors based on the specific characteristics of each scenario. Consequently, exclusive dependence on GNSS information is inadvisable due to its susceptibility to errors and instability under many practical operational situations. Recent advancements in vehicle state estimation have been driven by significant progress in computer vision. Modern approaches prioritize understanding ego-motion by leveraging external visual features [1]. For velocity estimation, the focus is on accurately tracking feature motion rather than determining their precise positions. For this reason, Optical Flow has been widely used in GNSS-denied tasks. Optical flow can arise from the relative motion of objects and the viewer. Consequently, Optical Flow can give important information about the spatial arrangement of the objects viewed and the rate of change of this arrangement [2,3]. In [4], the authors use an optical flow algorithm with a down-facing camera in an approximately flat outdoor or indoor scenario. In [5], the optical flow is computed using a Convolutional Neural Network and a Transformer, moving to the domain of Artificial Intelligence. However, Optical Flow techniques might suffer from well-known critical issues like occlusions, textureless regions, and inconsistency between the motion field and the optical flow itself (e.g., barber’s pole effect). In [6], the authors present a navigation system based on a down-facing Camera and IMU sensors. Due to cost and weight constraints, most solutions for aerial vehicles rely on 2D LiDARs for fast and accurate distance measurements in real time [7]. Despite hardware limitations, 2D LiDAR remains a pivotal component of velocity estimation algorithms. In Range Flow-based 2D Odometry (RF2O) [8], the authors set a minimization problem under the range flow constraint presented in [9]. Although RF2O employs an overdetermined system to mitigate occlusions and noise, the algorithm still experiences issues in certain corner cases, and it does not handle the degradations that might affect the point clouds, as described in [10,11]. Kinematic constraints are not the only possible way to determine the velocity vector from 2D LiDAR scans. Iterative Closest Point (ICP) is a pointcloud registration algorithm that provide the displacement estimation by solving an optimization problem on the transformation parameters, hence it is widely used for position and velocity estimation, as in [10,12]. Generalized-ICP (G-ICP) [13,14] defines a probabilistic framework to solve the scan matching problem and retrieve the transformation between two (eventually consecutive) LiDAR scans. However, this approach is even more sensitive to possible occlusions or exogenous changes in the scene and frequently encounters challenges related to motion degeneracy, particularly in geometrically similar environments. Currently, the teleoperation field lacks a reliable velocity estimator in GNSS-denied environment transition, and that could run on a low computational power and memory hardware set like the one on Single-Board Computers [15]. This velocity estimator should not require a detailed map of the environment, but, if possible, should still be able to benefit from information about loop closures. Our approach integrates a Sequential Kalman Filter (SKF) with visual landmarks, providing enhanced robustness against corner critical cases like temporary occlusions and dynamic elements, ensuring stable velocity estimation during GNSS-denied navigation. The proposed architecture directly addresses the issue of velocity estimation deterioration and divergence during transitions between outdoor and indoor environments. It introduces a streamlined yet effective pipeline that can be executed on the vehicle with minimal computational demand. The primary objective is to ensure accurate and stable velocity estimation, even in the absence of GNSS positioning data. The novelty and contributions of this paper can be summarized as follows: the proposed system is designed to deliver accurate and consistently bounded velocity estimations in GNSS-degraded or denied environments. It achieves this by utilizing relevant environmental features as beacons of opportunity to continuously track the motion of the vehicle in the environment. This capability, along with the option to maintain a database of these landmarks over time, not only improves positioning and velocity estimation but also offers valuable insights for addressing the loop closure problem. A key advantage of the system is its exclusive focus on velocity estimation, making it resilient to errors in landmark position estimations, provided these errors remain bounded and stable.

## 2. Materials and Methods

**Figure 1:**
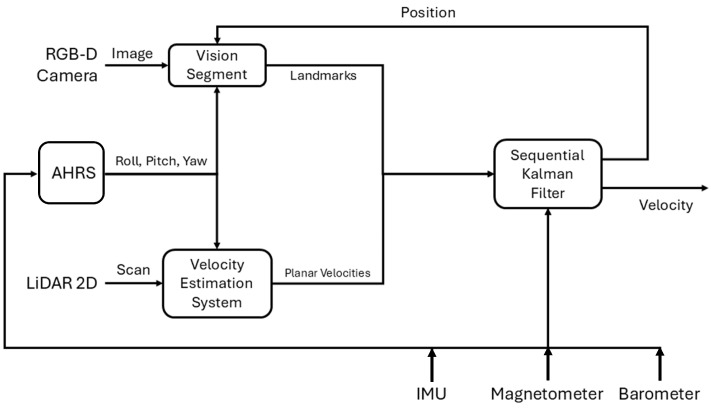
Velocity estimation pipeline.

In this section, the velocity estimation system structure (Figure 1) is exhaustively deepened, providing a comprehensive view of the pipeline. The proposed algorithm is based on the intuition that the velocity estimation can not diverge as long as the position of the localized features in view, and used as beacons of opportunity, has an error that remains bounded in the observation period. A boundedness analysis of the validity of the concept above can be found in the Appendix A.

### 2.1. Notation

The notation needed to explain the problem is defined as follows. Representing the camera frame as {c}, the navigation frame as {n}, and the body frame as {b}, we denote

${\tilde{P}}_{O_{i}}^{c}$: the *i*-th cluster position measured in the camera frame.
${\hat{P}}_{O_{i}}^{n}$: the estimated landmark position in the navigation frame.
$C_{b}^{c}\in SO\left(3\right)$: the body-camera rotation matrix.
$O_{c}^{b}$: a vector from the camera frame origin to *B*, the body frame origin.

In order to analyze the residual, the errors for both measurement and estimation vectors and rotation matrices are represented, respectively, with an additive and multiplicative model, that is (for generic frames *i* and *j*):

(1) $$\begin{array}{cc} {\tilde{P}}_{O_{i}}^{j} & =P_{O_{i}}^{j}+\delta P_{O_{i}}^{j},\\ {\tilde{C}}_{i}^{j} & =\left[I-\phi \wedge \right]C_{i}^{j} \end{array}$$

where $\phi \in R^{3}$ encodes attitude error, and $\delta P_{O_{i}}^{j}$ is the derivative error vector.

### 2.2. Vision Segment

**Figure 2:**
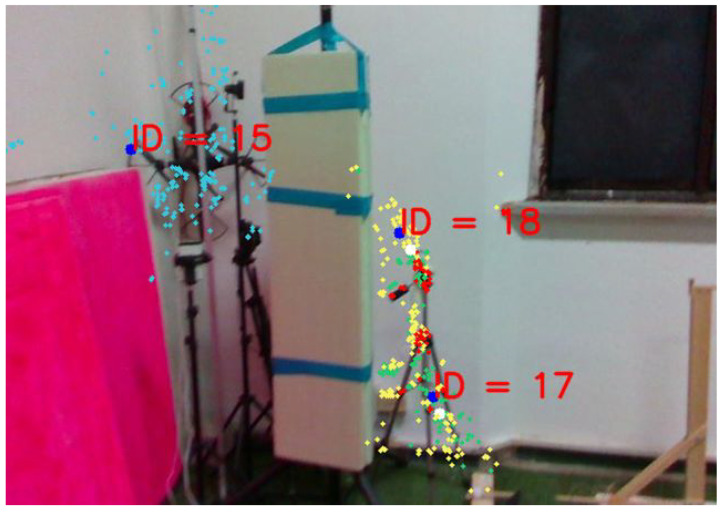
Camera capture with the relative feature extraction and clusterization. Each feature is represented by a dot and each landmark is represented by a colour and an ID.

The Vision Segment is responsible for creating and identifying opportunity landmarks, which are essential for generating position measurements within the Kalman Filter framework. To achieve this, the algorithm takes as input a depth image, an RGBD camera image, and the estimated position provided by the navigation filter. The RGB part of the image is processed using the off-the-shelf ORB feature detection algorithm [16]. This algorithm identifies key points within the image, referred to as features $f_{i}\left[u,v\right]$, where $f_{i}$ represents the ith feature in the 2D camera frame, with coordinates *u* and *v* expressed in pixels. Figure 2 shows a processed acquisition from the vision module, highlighting, as an example, three landmarks, each annotated with its corresponding identifier.

Assuming the pinhole camera model with intrinsic parameters $\left[\phi_{x},\phi_{y}\right]$ and $\left[c_{x},c_{y}\right]$, the 3D point $P_{f}^{C}$ in the camera frame associated with the feature *f* from the RGB image is defined as follows:

$$\begin{array}{cc} P_{f,X}^{c} & =\frac{(u-c_{x})Z}{\phi_{x}},\\ P_{f,Y}^{c} & =\frac{(v-c_{y})Z}{\phi_{y}},\\ P_{f,Z}^{c} & =Z. \end{array}$$

with the relative depth *Z* obtained from the depth component of the RGBD image. Using the vehicle’s position $P_{O_{cam}}^{n}$ and its own orientation $C_{c}^{n}$, we can compute the feature position in the navigation frame as follows:

(2) $$P_{f}^{n}=P_{O_{cam}}^{n}+C_{c}^{n}P_{f}^{c}.$$

In order to identify easily recognizable objects in the scene to be used later as landmarks for navigation, observed features undergo agglomerative clustering, utilizing a distance-based similarity measure through the application of the Hierarchical Density-Based Spatial Clustering of Applications with Noise (HDBSCAN) algorithm [17]. Once created, clusters are not immutable, though; they can be gradually enhanced by the integration of new features every time they are revisited by the camera’s vision algorithm. Once features are grouped into clusters, the cluster is assigned a fixed position that remains fixed, unaffected by localization errors from features added later. This approach helps prevent discontinuities in the vehicle’s odometry. The position of the *n*th cluster $O_{cluster}^{n}$ in navigation frame is first computed from the *k*th identified features $f_{i}$:

(3) $$O_{cluster}^{n}=\frac{\sum_{i=1}^{k}P_{f_{i}}^{n}}{k}$$

As anticipated, the cluster origin remains fixed, and each single constituent feature is stored as the distance δ from the center:

(4) $$\delta_{i}=P_{f}^{n}-O_{cluster}^{n}.$$

As the vehicle navigates in the environment surrounding the cluster, it may identify supplementary relevant features. When this happens, these features are dynamically incorporated into the cluster, leveraging the representation specified in (4). This is a peculiar property of this algorithm and has the fundamental advantage of keeping the cluster fixed in the navigation frame even when adding new features and refining its structure during motion. Unlike other approaches, like SLAM-based navigation algorithms, which usually try to improve the estimation of detected clusters/features in the environment, this approach guarantees that no drift will occur in detected features and clusters even in poor visibility situations. As a result, the cluster localization error, directly dependent on the navigation error at the time of cluster creation, does not drift, ensuring consistent velocity estimation as long as the cluster remains in view. This is the main purpose of the navigation system architecture described in this paper.

When a cluster has been seen for a certain amount of time, it is promoted to a landmark and used afterwards to generate position fixes for the navigation filter. Landmarks are clusters of features localized with respect to the navigation frame and are assumed to be fixed in space. Management of possibly moving clusters is an open issue that is not considered here, and will be the subject of future study. An overview of this process is described using pseudo-code in Algorithm 1.

The algorithm can be divided into three parallel threads:

- Features of the current image (*k*) are compared with those of the clusters formed at the previous iteration (k−1) that have not yet become a temporary cluster; if enough features match, a cluster is formed and is added to the set of temporary clusters.
- Features of the current image are compared with those of the set of temporary clusters; temporary clusters are clusters observed in the past but not yet promoted to landmarks. If a temporary cluster is again in view, a counter is increased; when it passes a predefined threshold, the cluster becomes a landmark.
- Clusters of the current image (at time *k*) are compared with the features of all previously found landmarks that are potentially in view. The HDBSCAN descriptors are used to match clusters of the current image with the previously found landmarks, potentially in view. This allows the recognition of landmarks, the refinement of their structure by adding additional previously unseen features to them, and the determination of their positions in the camera frame.

Upon becoming a landmark, a temporary cluster is given a position in the navigation frame, calculated as the average of the positions of the cluster points, which remains immutable in time. When a landmark is in view, the vehicle’s position fix given as input to the Sequential Kalman Filter ${\tilde{P}}_{O_{cam}}^{n}$ is obtained from the landmark as

(5) $${\tilde{P}}_{O_{cam}}^{n}=O_{cluster}^{n}+\frac{\sum_{i=1}^{m}\left(\delta_{i}-C_{c}^{n}\;P_{f_{i}}^{c}\right)}{m}$$

where $O_{cluster}^{n}$ is the stored cluster center, $\delta_{i}$ is the stored offset of *i*th feature from the center, and $P_{f_{i}}^{c}$ is the current position in camera frame of *i*th detected feature at the current time belonging to the cluster.
**Algorithm 1** Cluster Handling Algorithm# Parameters${\mathrm{threshold}}_{\Lambda }=90$ #90 frames, 3 s in view**Input:** RGB Image *I***Output:** Landmark Set Λ**# Extract and cluster features from current image**$F_{current}\leftarrow \mathrm{ORB}\left(I\right)$$C_{current}\leftarrow \mathrm{HDBSCAN}\left(F_{current}\right)$**# Step 1: Match with previous clusters**$C_{temp}\leftarrow \emptyset$**for** each cluster *c* in $C_{prev}$ **do**      **if** $\mathrm{Match}(F_{current},c.\mathrm{features})$ **then**            $C_{temp}.\mathrm{add}\left(c\right)$**# Step 2: Update temporary cluster view counts****for** each cluster *c* in $C_{temp}$ **do**      **if** $\mathrm{Match}(F_{current},c.\mathrm{features})$ **then**            c.viewCount←c.viewCount+1      **if** $(c.\mathrm{viewCount}>{\mathrm{threshold}}_{\Lambda }$a) **then**            Λ.append(c)**# Step 3: Long-Term Landmark Update**$\left[i_{cluster},i_{landmark}\right]\leftarrow \mathrm{Match}\left(C_{current},\Lambda \right)$**for** k=1 to $|i_{cluster}|$ **do**      $idx_{c}\leftarrow i_{cluster}\left[k\right]$      $idx_{\lambda }\leftarrow i_{landmark}\left[k\right]$      $F_{new}\leftarrow C_{current}\left[idx_{c}\right].\mathrm{features}\backslash \Lambda \left[idx_{\lambda }\right].\mathrm{features}$      $\Lambda \left[idx_{\lambda }\right].\mathrm{add}\left(F_{new}\right)$

### 2.3. Sequential Kalman Filter

The core of the system is made up of a Sequential Kalman Filter (SKF) that provides the final state estimation [18]. The state of the system is represented as

(6) $$x={\left[p_{x},v_{x},a_{x},p_{y},v_{y},a_{y},p_{z},v_{z},a_{z}\right]}^{T}$$

where $p=[p_{x},p_{y},p_{z}]$ represents the vehicle position, $v=[v_{x},v_{y},v_{z}]$ is the vehicle velocity and $a=[a_{x},a_{y},a_{z}]$ is the vehicle acceleration. The choice to use acceleration as state variables, and thus acceleration as a measurement to be employed during the SKF update step and not as system input, allows both to filter out accelerometer noise, and to take into account possible attitude estimation errors. The SKF, as the standark Kalman Filter, runs both the prediction and the correction steps in a consecutive fashion. The prediction step is computed as in Equation (7) and correction as in (8).

(7) $$\left\{\begin{array}{c} x_{k+1|k}=Ax_{k|k}+Bu\\ P_{k+1|k}=AP_{k_{k}}A^{T}+Q \end{array}\right.\;.$$

(8) $$\left\{\begin{array}{c} K=PC^{T}{\left(R+CPC^{T}\right)}^{-1}\\ x_{k+1|k+1}=x_{k+1|k}+K\left(y-Cx\right)\\ P_{k+1|k+1}=\left(I-KC\right)P_{k+1|k} \end{array}\right.\;.$$

where *P* is the covariance matrix, *K* is the Kalman gain matrix, and *C* is the observation matrix.

The discrete-time dynamic is represented as

$$x_{k+1}=Ax_{k}$$

with

(9) $$\begin{array}{cccc} A=\left[\begin{array}{ccc} M & 0_{3\times 3} & 0_{3\times 3}\\ 0_{3\times 3} & M & 0_{3\times 3}\\ 0_{3\times 3} & 0_{3\times 3} & M \end{array}\right]\; & \; & & M=\left[\begin{array}{ccc} 1 & dt & 0.5dt^{2}\\ 0 & 1 & dt\\ 0 & 0 & 1 \end{array}\right]. \end{array}$$

The update step is performed using a sequential Kalman Filter approach [19], and thus depends on the number of data sources providing information: an update step is performed for each landmark in sight since it is possible to incorporate each measurement information into the update filter step sequentially and independently, as demonstrated in [19]. The SKF can integrate data from supplementary sensors, such as the IMU and barometer, which, when included, enhance the precision and robustness of the vehicle’s state estimation. The use of the SKF sequential updates method allows to reduce the computational cost since it does not require the inversion of large and time and size-varying matrices, regardless of the current number of landmarks in view. In particular, when the system reads from the Velocity Estimation algorithm, the matrix *C* is

$$C_{vel}=\left[\begin{array}{ccccccccc} 0 & 1 & 0 & 0 & 0 & 0 & 0 & 0 & 0\\ 0 & 0 & 0 & 0 & 1 & 0 & 0 & 0 & 0 \end{array}\right]$$

And when it gets the camera landmark positions

$$C_{cam}=\left[\begin{array}{ccccccccc} 1 & 0 & 0 & 0 & 0 & 0 & 0 & 0 & 0\\ 0 & 0 & 0 & 1 & 0 & 0 & 0 & 0 & 0\\ 0 & 0 & 0 & 0 & 0 & 0 & 1 & 0 & 0 \end{array}\right].$$

As shown in Figure 1, the possible measurements are IMU, Attitude and Heading Reference System (AHRS), the fixes provided by the vision segment (discussed in Section 2.2) and 2D LiDAR scans that are processed through a 2D LiDAR velocity estimator. The stochastic characteristics of the filter are defined by the process and measurement noise covariance parameters, which are summarized in Table 1.

## 3. Experiments and Results

**Figure 3:**
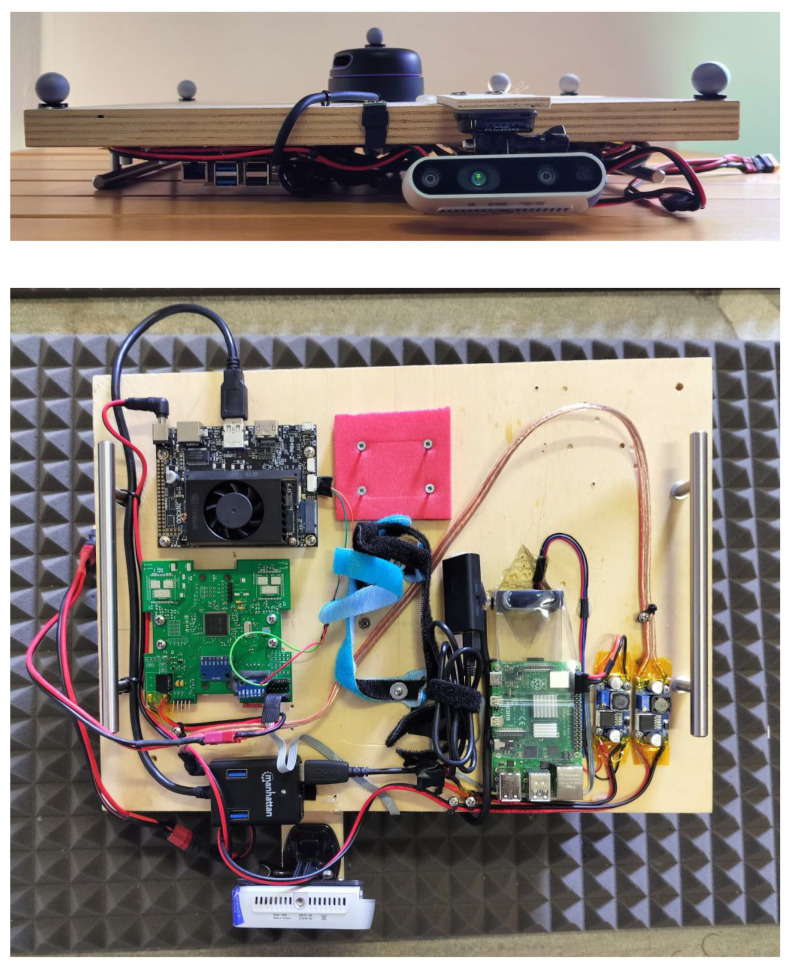
Platform front view (**Up**) with a RP A3 Lidar and an Intel D435 Depth Camera, bottom view (**Down**) with the NVIDIA Xavier and IMU.

In order to evaluate the potential of the proposed method for remotely piloting a vehicle, a dummy vehicle was constructed (refer to Figure 3), equipped with the same sensors and computing power as the real quadcopter Icaro Buzz, suitable for indoor inspection. This includes the Icaro Autopilot, a single-plane LiDAR sensor (specifically, a Slamtec RPLidar A3 (Slamtec Co., Ltd., Shanghai, China) [20], an NVIDIA Jetson Xavier (NVIDIA Corporation, Santa Clara, CA, USA), an Intel D435 RGBD camera (Intel Corporation, Santa Clara, CA, USA) that feeds both the RGB and the depth image to the system. During tests, a motion capture system by Vicon (VMC) was used to generate groundtruth data with an accuracy of up to 0.25 mm [21]. All sensor data are exchanged through ROS (Robot Operating System) communication network. Each onboard sensor publishes its measurements on dedicated ROS topics, to which the onboard computer subscribes. The received measurements are synchronized using a common time base and processed within the Sequential Kalman Filter (SKF) described in Section REF. The filter updates the vehicle state estimate at a fixed frequency of 50 Hz. The overall system architecture is fully integrated within the ROS framework, enabling modular sensor data acquisition and real-time state estimation while maintaining low computational overhead suitable for onboard execution.

The velocity remains in the range [−0.5,0.5] m/s in all experiments to mimic an environment exploration scenario where the remote pilot conducts an initial slow velocity survey. This velocity range not only reflects the specific application for which the system has been developed but also grants LiDAR the ability to work in an optimal operational range. Consequently, after an evaluation of the filter performance in different planar motions, the tests concentrate on specific cases that may hinder the effectiveness of LiDAR odometry and sensor fusion assistance, such as moving objects or temporal occlusions that negatively impact odometry using the LiDAR. Four different experiments are presented: The first two demonstrate the overall system performance in a planar exploration scenario without GNSS. The third experiment assesses the system’s performance during a transition from a GNSS-enabled environment to a GNSS-denied one. The final experiment shows how the filter behaves in situations involving complex data degradation conditions. During the experimental phase, the average processing time of the Vision Segment callback, responsible for feature extraction and for updating the cluster and landmark databases, was approximately 25 ms per camera frame. Since this processing time is shorter than the interval between consecutive image acquisitions, the Vision Segment is able to process each frame before the arrival of the next one, thus maintaining near-real-time execution. The inclusion of the Vision Segment requires approximately 350 MB of memory on the onboard computer during runtime and operates entirely on the CPU, without GPU acceleration. The proposed architecture achieves a substantially lower computational and memory footprint with respect to standard SLAM or LIVO systems [22,23] by avoiding global mapping and nonlinear optimization, enabling fully CPU-based execution with bounded per-frame complexity while preserving reliable velocity estimation. This design choise preserves GPU availability for other tasks and further supports deployment and directly supports the experimental objective of maintaining stable, low-latency velocity estimates during GNSS-degraded operation, which is critical for remote piloting and control performance rather than long-term global consistency.

### 3.1. First Experiment: Linear Motion

**Figure 4:**
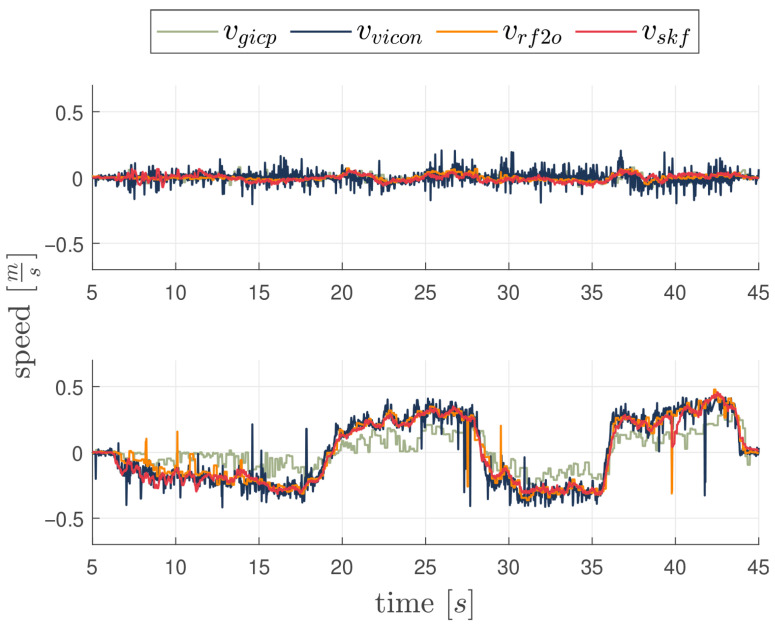
Velocity estimation from RF2O, G-ICP, RF2O-enabled SKF and groundtruth for the first experiment. X-Axis on top and Y-Axis on the bottom side.

**Figure 5:**
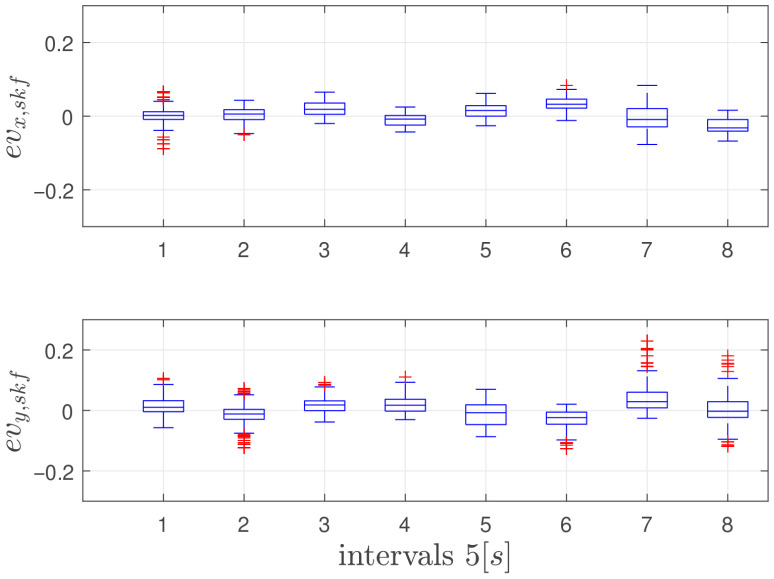
Box Plot of the error between SKF Estimated and VMC Velocity in the first experiment. Each box refers to a 5 s time frame and red signs represent the outliers.

In this experiment, the vehicle moves at a height of 0.7 m from the ground, moving back and forth from its original position along the y-axis in the navigation frame by a total distance of 6 m with a peak velocity of 0.5 m/s. The lateral movement allows for evaluating the feature tracking algorithm’s performance, as the camera maintains a consistent view of the same features throughout the entire movement. The SKF does not use the VMC signal and relies only on fixes from landmarks for position and velocity estimation. The actual VMC estimated velocity is only used as ground truth. In order to asses the performances of the proposed architecture, SKF estimation is compared with two state of the art LiDAR based velocity estimation algorithms. Such are RF2O and G-ICP. To facilitate a direct comparison between the LiDAR velocity estimation and the SKF output, the velocity estimation filter module employed is the algorithm that demonstrated superior performance in the proposed trajectories, specifically the RF2O algorithm. Figure 4 shows true and estimated velocities along the X and Y axes, and Figure 5 shows statistics of velocity estimation error with boxplots showing median, 25 and 75 percentiles, and data considered outliers in the area outside the whiskers. Each element of the plot represents statistics of a 5 s time frame. As can be seen, the velocity estimation error does not grow significantly in time, as it should be expected from a pure inertial system; furthermore, the mean velocity error remains approximately zero, supporting the conclusion that this navigation system can be effectively used as feedback for the vehicle velocity controllers without generating dangerous drifts. Oscillations in velocity error statistics during the flight are mainly due to varying visibility conditions of the landmarks. Performance indexes of velocity errors are listed in Table 2. As expected, the performances of the SKF algorithm demonstrate marginal improvements over both G-ICP and RF2O, in particular over the y-axis, which is the axis of principal motion.

### 3.2. Second Experiment: Planar Motion

**Figure 6:**
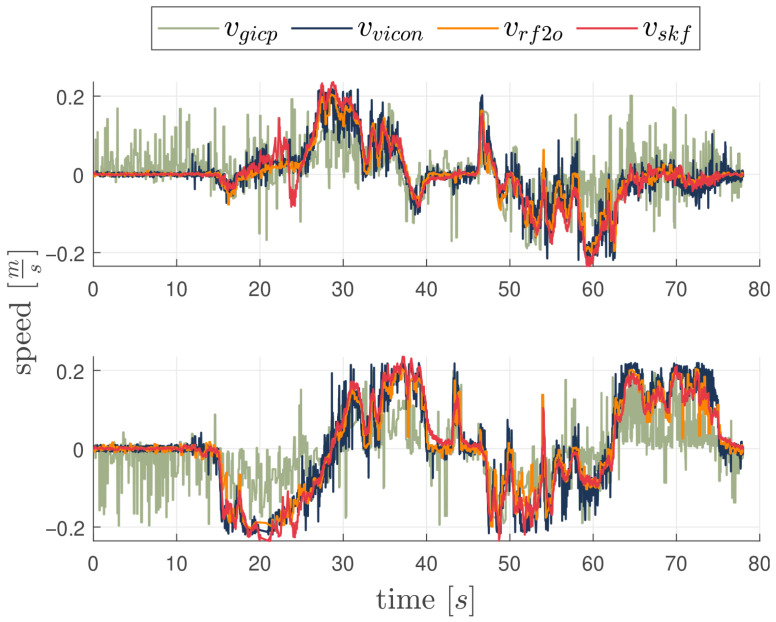
Velocity Estimation from RF2O, G-ICP, RF2O-enabled SKF and groundtruth for the second experiment. X-Axis on top and Y-Axis on the bottom side.

**Figure 7:**
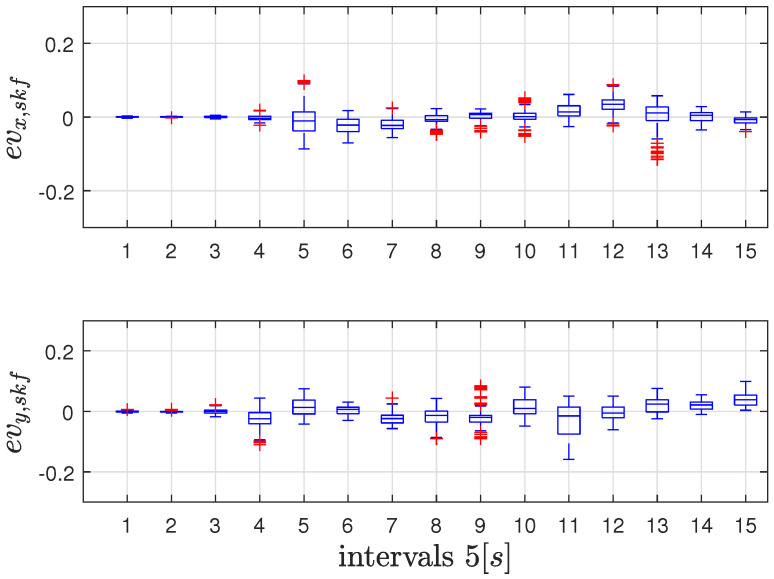
Box Plot of the error between SKF Estimated and VMC Velocity in the second experiment. Each box refers to a 5 s time frame and red signs represent the outliers.

This experiment assesses the system performance in a planar motion on the xy-plane. The vehicle moves on an approximately semicircular path with a diameter of 2 m at a constant altitude of 0.7 m. The SKF uses again RF2O as velocity measure because of its accuracy with respect to G-ICP. Performance are presented in Figure 6 and Figure 7 and the results in Table 3. The SKF does not use the VMC signal and relies only on fixes from landmarks for position and velocity estimation. The actual VMC estimated velocity is only used as ground truth. Also in this case velocity errors remain small and do not drift in time. The results proves that the addition of the vision module improves even if still marginally the Lidar-based velocity estimation; the performance of the SKF filter and RF2O is again comparable, even if, both the global indexes $\mu_{x},\mu_{y}$ and the standard deviation $\sigma_{x},\sigma_{y}$ show that SKF improves the RF2O estimation.

### 3.3. Third Experiment: Loop Closures

In the third experiment, the vehicle moves at a constant altitude of 0.7 m inside the laboratory volume, a feature-rich environment, where the VMC signal is available. The exploration starts inside the laboratory, where the vehicle can benefit from a feature-dense environment and position measurements from VMC. After 10 s, the navigation filter stops using information from the VMC, and after a short flight in the laboratory, the vehicle moves to a feature-poor environment, specifically a hallway, where, additionally, the VMC signal is no longer available; thus, no ground truth is available. Following a brief survey of this new area, the vehicle returns to the area covered by the VMC system. The primary objective of this experiment was to assess, at least qualitatively, the capability of the algorithm to keep position and velocity errors low for prolonged periods of time when exploring complex environments and to evaluate the navigation system’s capacity to recover its position and velocity estimates by reacquiring previously mapped long-term landmarks.

**Figure 8:**
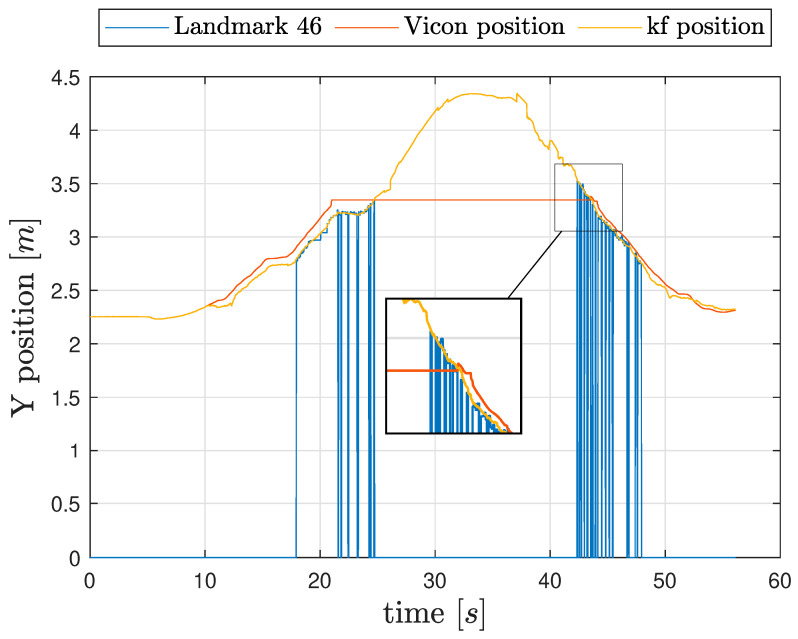
Third experiment: True (red) and estimated (orange) position in the fourth experiment (y-axis only). Landmark 46 fixes detections are drawn to show the little accumulated position error during the simulated flight outside the VMC area. Blue spikes show when the landmark recognition happens.

In order to evaluate the navigation performance both estimated velocity and estimated position will be compared. During exploration of the laboratory several clusters of features were promoted to landmark, and thus used as local position fixes; Figure 8 shows the vehicle position (y-axis only) as measured by the VMC where available, estimated by the navigation filter, and estimated using Equation (5) from one of the landmarks: Landmark 46 was chosen for this example since it is in plain view from t≈18 s to t≈25 s, just before exiting the area covered by VMC. When the cluster is not in view, the cluster position is shown as 0.

The zoomed-in portion of the figure shows that when the marker comes back into view, at approximately t=42 s, the navigation filter estimated position matches closely the estimated position from the landmark, suggesting that the SKF position estimate has not accumulated a significant error, even when navigating in a feature-poor environment, indicating that the mean estimated velocity error, from t≈25 s to t≈42 s was very close to 0.

**Figure 9:**
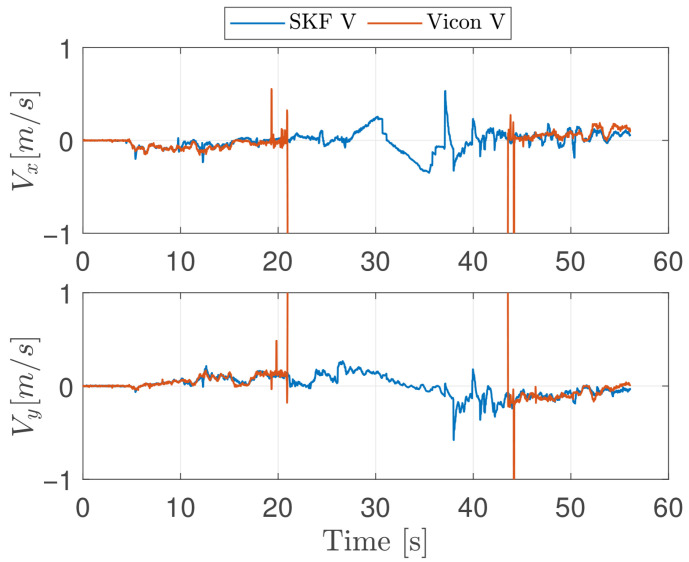
Velocity Estimation and groundtruth in the third experiment: VMC cutoff happens after 21 s. Groundtruth is not available as long as the vehicle is outside the VMC area.

Figure 9 shows the estimated and groundtruth (from VMC) velocity for this experiment. Velocity (and position) groundtruth is lost, when entering the hallway, at t≈21 s and recovered, when returning inside the laboratory, at t≈42 s; It is clearly not possible to compute an estimation error in the period without groundtruth but the analysis above indicates that velocity estimation should have been good also during the period without ground truth, otherwise a drift should have been observed in position estimates.

To conclude, even if small, the contribution of the intrinsic loop closure capability of the algorithm can also be observed.

### 3.4. Fourth Experiment: Corner Cases

**Figure 10:**
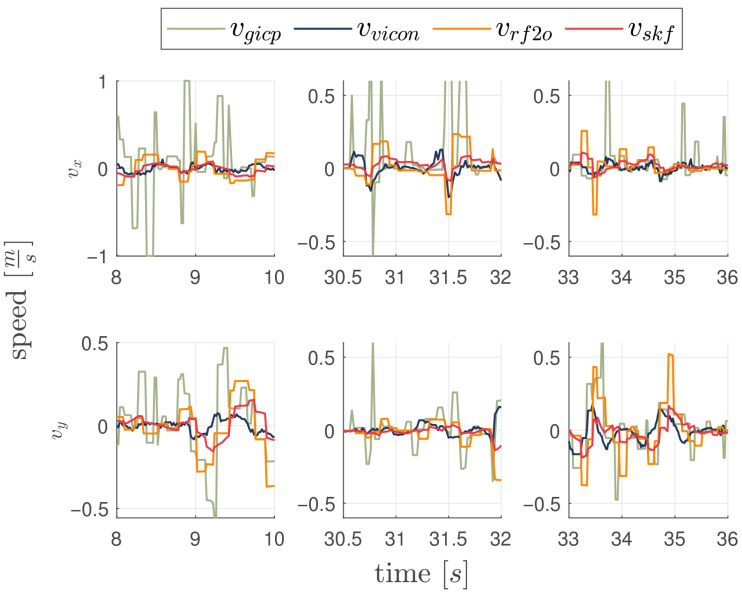
Velocity estimators response to three different perturbations in LiDAR scans, that are occlusions and impulsive disturbances. Each column shows velocity on the X (**top**) and Y (**bottom**) axes for each occlusion.

A vehicle employing a LiDAR odometry algorithm for velocity estimation is subject to limitations caused by occlusions or dynamic objects in the navigation environment [8]. In this set of experiments, the robustness of the proposed velocity estimation algorithm is evaluated under conditions of temporary LiDAR-only velocity estimation occlusion degradation. Specifically, occlusions were deliberately and abruptly introduced three times during testing by momentarily placing a panel in front of the LiDAR sensor and then removing it while the vehicle is stationary. The resulting degraded measurements introduce spikes and errors in the LIDAR-based algorithms’ velocity estimates. Figure 10 depicts the system’s performance under such conditions, emphasizing the capacity of the proposed filter to compensate for errors arising in the RF2O estimation. The figure shows the velocity estimates across three successive occlusion events, where the LiDAR-only odometry algorithm (orange, green) experiences the expected degradation. Each event is represented as a column comprising velocity estimates from VMC, RF2O, GICP, and the proposed algorithm (SKF), with the upper subplots corresponding to the x-axis velocity and the lower subplots to the y-axis velocity. In the first and third events, degradation is primarily observed along the y-axis, whereas in the second event the effect is more pronounced along the x-axis. Table 4 supports the observations by reporting the mean, standard deviation, and mean squared error of the velocity estimates during each occlusion event. Across all three experiments, the proposed SKF method consistently achieves the lowest mean squared error, with values ranging from 0.0757 to 0.0893, lower than those obtained by RF2O and G-ICP. The proposed algorithm also presents smaller deviations from the true velocity. In contrast, RF2O and G-ICP exhibit larger mean errors and higher variability, particularly during the first and second occlusions. These results confirm the superior robustness of the SKF algorithm in mitigating the adverse effects of temporary LiDAR occlusions.

## 4. Limitations and Future Works

In the previous sections, we introduced the conceptual foundations of the proposed system and provided a detailed description of the vision module, which constitutes the main contribution of this work in the specific context of navigation under GNSS-degraded or GNSS-denied conditions. The proposed addition demonstrates a consistent improvement in overall system performance, not only in the complete absence of GNSS (Experiments 1 and 2) and during GNSS signal loss (Experiment 3), which represents the primary focus of this work, but also as an effective support mechanism during temporary failures of LiDAR odometry (Experiment 4). While the proposed navigation architecture demonstrates robust and bounded velocity estimation in GNSS-degraded and GNSS-denied environments, several limitations must be acknowledged. These limitations also highlight clear and relevant directions for future research. First, the current vision-based landmark management strategy assumes a predominantly static environment. Clusters promoted to landmarks are treated as fixed in the navigation frame, and the presence of moving or semi-static objects is only partially addressed through the landmark promotion policy, whereby clusters are promoted only if they remain observable for a predefined number of consecutive frames. This strategy enables the rejection of clearly dynamic elements, such as moving vehicles, pedestrians, or machinery; however, it may still lead to incorrect landmark associations in highly dynamic scenes, potentially degrading the state estimate. Future work will focus on incorporating explicit landmark validation and rejection mechanisms, as well as dynamic landmark classification strategies, to improve robustness in environments with significant motion. Second, experimental validation assumes low-acceleration maneuvers with a maximum speed of approximately 0.5 m/s. This choice reflects typical remote piloting and inspection scenarios and ensures operation within the optimal range of the LiDAR sensor. While well aligned with the target application, this constraint limits the ability to draw conclusions about system performance under higher speeds, aggressive maneuvers, or high-angular-rate motions. Future experimental campaigns will extend the evaluation to more demanding dynamic regimes in order to assess scalability to faster platforms and more challenging operational profiles. Third, the system relies on sufficient visual texture and feature richness to initialize and maintain landmarks of opportunity. In environments that are both visually feature-poor and affected by LiDAR degradation, such as long uniform corridors or large open spaces with sparse geometric structure, the availability of reliable landmarks may be limited. In such cases, prolonged perception degradation may negatively impact estimation quality. Future developments will investigate adaptive sensor weighting strategies and tighter integration with inertial cues to mitigate these effects. Additionally, global appearance plays a critical role in camera-based perception: abrupt illumination changes or low-light conditions significantly degrade feature detection and matching performance. In this work, experiments were conducted under static lighting conditions to isolate and evaluate the contribution of the vision module in mitigating GNSS degradation. A natural direction for future work is to enhance the robustness of the feature extraction pipeline to varying illumination conditions. Finally, although the Sequential Kalman Filter formulation provides computational efficiency and stable estimation, filter tuning parameters were empirically selected and kept fixed across all experiments. While this approach simplifies deployment, adaptive covariance tuning or consistency-monitoring mechanisms could further improve robustness, particularly during sensor degradation events or abrupt environmental transitions. Overall, addressing these limitations will broaden the applicability of the proposed architecture beyond controlled inspection scenarios, enabling more autonomous, resilient, and long-duration operations in complex and unstructured environments.

## 5. Conclusions

This paper presented the theory and experimental results of a navigation system specifically designed for accurate non-drifting velocity estimation and with much less emphasis on position estimation. The vision system allows to handle transitions into a GNSS-denied environment by creating landmarks of opportunity during the exploration; this helps both to limit velocity error and to serve as loop closure when the landmarks become visible again. Various experiments were conducted to assess overall performance and validate the effectiveness of the proposed solution. The results indicate that the architecture is well-suited to the previously mentioned problem and shows promising performance. This is evidenced by the fact that every velocity estimation error index, especially the Mean Error, is in the order of a few millimeters per second.

## Figures and Tables

**Table 1:** Process and measurement noise covariance parameters used in the SKF.

Matrix	Diagonal Elements
*Q*	$\left[10^{-8},\;10^{-1},\;10^{-2},\;10^{-8},\;10^{-1},\;10^{-2},\;10^{-5},\;10^{-4},\;10^{-2}\right]$
$R_{\mathrm{GPS}}$	$\left[10^{3},\;10^{3},\;10^{4},\;10^{4},\;3\cdot 10^{3}\right]$
$R_{\mathrm{CAMERA}}$	$\left[10^{2},\;10^{2},\;10^{3}\right]$
$R_{\mathrm{IMU}}$	[1,1,0.9]
$R_{\mathrm{LiDAR}}$	$\left[10^{5},\;10^{5}\right]$
$r_{baro}$	[0.85]

**Table 2:** First experiment results. Indexes are: $\mu_{x,y}$ mean on x and y, $\sigma_{x,y}$ standard deviation on x and y, and Mean Squared Error (mse). Bold values highlight the best results.

Algorithm	$\mu_{x}$	$\mu_{y}$	$\sigma_{x}$	$\sigma_{y}$	mse
SKF	0.0035847	**0.0052489**	0.028217	**0.042968**	**0.0026815**
G-ICP	0.0007491	−0.0081371	0.026469	0.15074	0.023478
RF2O	**0.00060497**	−0.0079874	**0.023929**	0.048903	0.0030267

**Table 3:** Second experiment results. Indexes are: $\mu_{x,y}$ mean on x and y, $\sigma_{x,y}$ standard deviation on x and y, and Mean Squared Error (mse). Bold values highlight the best results.

Algorithm	$\mu_{x}$	$\mu_{y}$	$\sigma_{x}$	$\sigma_{y}$	mse
SKF	**0.00026773**	**−0.0010225**	0.024098	**0.032205**	**0.0016186**
G-ICP	−0.0084467	0.00418	0.058429	0.10314	0.014138
RF2O	0.0010359	−0.0014562	**0.019923**	0.038378	0.0018725

**Table 4:** Occlusion experiment results. Indexes are: $\mu_{x,y}$ mean on x and y, $\sigma_{x,y}$ standard deviation on x and y, and Mean Squared Error (mse). Bold values highlight the best results.

**(a) First occlusion**					
**Algorithm**	$\mu_{x}$	$\mu_{y}$	$\sigma_{x}$	$\sigma_{y}$	**mse**
SKF	**0.0088627**	**−0.0091305**	**0.051895**	**0.08442**	**0.081612**
G-ICP	−0.058464	−0.042017	0.20318	0.31524	0.26984
RF2O	−0.0050858	0.011968	0.12108	0.14796	0.16283
**(b) Second occlusion**					
**Algorithm**	$\mu_{x}$	$\mu_{y}$	$\sigma_{x}$	$\sigma_{y}$	**mse**
SKF	−0.031128	0.015867	**0.053684**	**0.067324**	**0.075745**
G-ICP	−0.13207	**0.0029035**	0.35275	0.22401	0.24328
RF2O	**−0.01784**	0.022458	0.12309	0.11719	0.13059
**(c) Third occlusion**					
**Algorithm**	$\mu_{x}$	$\mu_{y}$	$\sigma_{x}$	$\sigma_{y}$	**mse**
SKF	−0.017734	**0.0050868**	**0.040857**	**0.097087**	**0.089323**
G-ICP	−0.049228	−0.040686	0.17659	0.22846	0.17242
RF2O	**−0.016114**	−0.010856	0.080844	0.17644	0.145

**Figure A1:**
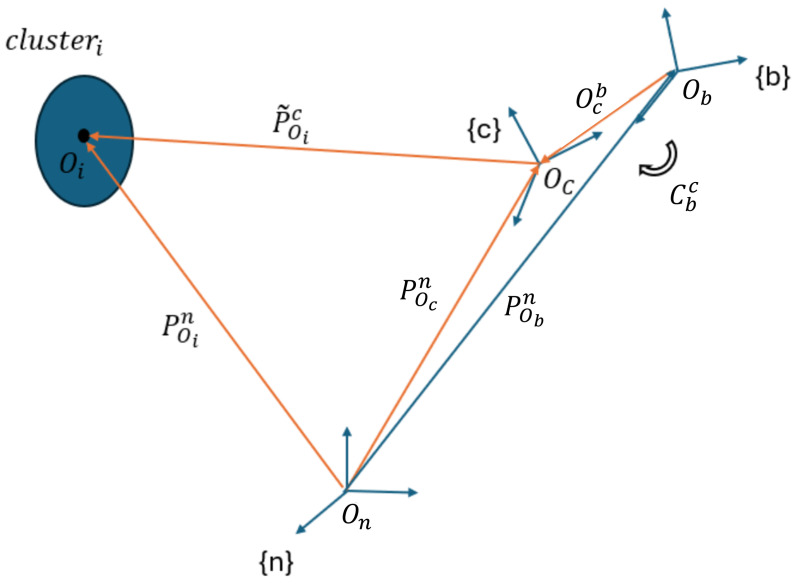
Reference systems scheme.

## Data Availability

The raw data supporting the conclusions of this article will be made available by the authors on request.

## Appendix A

This section presents a mathematical analysis of position estimation error to support its boundedness and, consequently, that of velocity estimation error. The following result represents a pivotal element for the proposed architecture, as stated at the beginning of Section 2.

Using the previous notation, it is possible to express the *i*-th cluster position in the navigation frame as shown in Figure A1:

(A1) $${\hat{P}}_{O_{i}}^{n}={\hat{P}}_{O_{c}}^{n}+{\hat{C}}_{c}^{n}{\tilde{P}}_{O_{i}}^{c}.$$

where

(A2) $$\begin{array}{cc} {\hat{P}}_{O_{c}}^{n} & ={\hat{P}}_{O_{b}}^{n}+{\hat{C}}_{b}^{n}O_{c}^{b},\\ {\hat{C}}_{c}^{n} & ={\hat{C}}_{b}^{n}C_{c}^{b}. \end{array}$$

or equivalently:

(A3) $${\hat{P}}_{O_{i}}^{n}={\hat{P}}_{O_{b}}^{n}+{\hat{C}}_{b}^{n}{\hat{O}}_{c}^{b}+{\hat{C}}_{b}^{n}{\hat{C}}_{c}^{b}{\tilde{P}}_{O_{i}}^{c}.$$

Using the error models in (1) and removing second order terms, at the *k*-th instant, Equation (A3) becomes:

(A4) $$\begin{array}{cc} {\hat{P}}_{O_{i}}^{n} & =P_{O_{i}}^{n}+\delta P_{O_{b}}^{n}\left(k\right)+\Phi \left(k\right)\wedge C_{c}^{n}P_{O_{i}}^{c}\\ \; & \;+C_{b}^{n}C_{c}^{b}\delta P_{O_{i}}\left(k\right). \end{array}$$

where $\delta P_{O_{i}}\left(k\right)$ is the cluster localization error in camera frame due to the vision pipeline measurement error, $\delta P_{O_{b}}^{n}\left(k\right)$ is vehicle position estimation (navigation) error, Φ(k) is the vehicle attitude (navigation) estimation error, and time (k) represents the time the cluster is located for the very first time. Equation (A4) shows how the cluster position, fixed and defined when it is promoted to Landmark, is affected by the navigation and the camera measurement errors at that time. This error cannot be reduced in the future but, at the same time, will not increase either, thus, as long as this cluster is used to generate position fixes, the estimated vehicle position will not drift, yielding a close-to-zero mean velocity estimation error. When, at the generic instant k+N, the camera sees the cluster again, its predicted position in camera frame can be obtained with

(A5) $$\begin{array}{cc} \; & {\hat{P}}_{O_{i}\mathrm{pred}}^{c}={\left({\hat{C}}_{c}^{n}\right)}^{T}\left(k+N\right)[{\hat{P}}_{O_{i}}^{n}\left(k\right)\\ \; & \;-{\hat{P}}_{O_{b}}^{n}\left(k+N\right)-{\hat{C}}_{b}^{n}\left(k+N\right)O_{c}^{b}]. \end{array}$$

and the residual fed to the KF: the difference between its predicted position and its measured position ${\tilde{P}}_{O_{i}}^{c}\left(k+N\right)$, is

(A6) $$R^{c}\left(k+N\right)={\tilde{P}}_{O_{i}}^{c}\left(k+N\right)-P_{O_{i}\mathrm{pred}}\left(k+N\right).$$

Using the error models in Equation (1), and neglecting higher order terms, Equation (A6) becomes

(A7) $$\begin{array}{cc} \; & R^{c}\left(k+N\right)={\tilde{P}}_{O_{i}}^{c}\left(k+N\right)-P_{O_{i}\mathrm{pred}}\left(k+N\right)=\\ \; & P_{O_{i}}^{c}\left(k+N\right)+\delta P_{O_{i}}^{c}\left(k+N\right)-C_{n}^{c}\left(k+N\right)\left[I+\Phi \left(k+N\right)\right]\\ \; & \;[{\hat{P}}_{O_{i}}^{n}\left(k\right)+\delta P_{O_{b}}^{n}-\Phi \left(k\right)\wedge C_{c}^{n}P_{O_{i}}^{c}+C_{c}^{n}\delta P_{O_{i}}^{c}\left(k\right)\\ \; & \;-P_{O_{b}}^{n}\left(k+N\right)-\delta P_{O_{b}}^{n}\left(k+N\right)\\ \; & \;+\left[I-\Phi \left(k+N\right)\wedge \right]C_{b}^{n}\left(k+N\right)O_{c}^{b}]. \end{array}$$

The residual in Equation (A7) can be rotated into navigation frame for a better analysis obtaining

(A8) $$\begin{array}{cc} \; & C_{c}^{n}\left(k+N\right)R^{c}\left(k+N\right)\approx \\ \; & \;\underset{\Delta P_{O_{b}}^{n}}{\underset{︸}{\delta P_{O_{b}}^{n}\left(k+N\right)-\delta P_{O_{b}}^{n}\left(k\right)}}+\\ \; & \;\underset{\Delta P_{O_{i}}^{n}}{\underset{︸}{\delta P_{O_{i}}^{n}\left(k+N\right)-\delta P_{O_{i}}^{n}\left(k\right)}}+\\ \; & \;\underset{\Phi P_{O_{i}}^{c}}{\underset{︸}{-\Phi \left(k+N\right)\wedge C_{c}^{n}\left(k+N\right)P_{O_{i}}^{c}\left(k+N\right)+}}\\ \; & \;\underset{\Phi P_{O_{i}}^{c}}{\underset{︸}{\Phi \left(k\right)\wedge C_{c}^{n}\left(k\right)P_{O_{i}}^{c}\left(k\right)}}. \end{array}$$

where:

- $\Delta P_{O_{b}}^{n}$ is the difference between navigation errors at times *k* and (k+N).
- $\Delta P_{O_{i}}^{n}$ is the difference between camera (vision-pipeline) measurement errors at times *k* and (k+N); this error is bounded under the realistic assumption that camera error does not diverge.
- the two terms $\Phi P_{O_{i}}^{c}$ together model the effect of attitude estimation error on the cluster position; given that attitude estimation error can be considered bounded, and terms $P_{O_{i}}^{c}\left(\cdot \right)$ are also bounded, these terms also are bounded.

Analysis of the above equation shows that, since stability of the Extended Kalman Filter keeps $R^{c}\left(k+N\right)$ bounded [24], and all other terms can be shown to be bounded under reasonable assumptions, then also the term $\Delta P_{O_{b}}^{n}$ is bounded. Boundedness of position navigation error is thus guaranteed as long as the same cluster is in view. When no cluster is in view or when, due to vehicle motion, past clusters are no more in view, position error may increase but divergence should be very slow, as demonstrated in the experimental tests. Ultimately, this guarantees, that with the possible exception of cluster switch times, the estimated velocity error should be approximately zero mean. This is the main goal of the proposed navigation filter.
